# Closer to the Reality—Proteome Changes Evoked by Endometrial Scratching in Fertile Females

**DOI:** 10.3390/ijms241713577

**Published:** 2023-09-01

**Authors:** Iwona Scheliga, Dunja M. Baston-Buest, Gereon Poschmann, Kai Stuehler, Jan-Steffen Kruessel, Alexandra P. Bielfeld

**Affiliations:** 1 Department of OB/GYN and REI (UniKiD), Medical Faculty and University Hospital Duesseldorf, Heinrich-Heine University, 40255 Duesseldorf, Germany; iwona.scheliga@med.uni-duesseldorf.de (I.S.); jan-steffen.kruessel@med.uni-duesseldorf.de (J.-S.K.); 2 Institute for Molecular Medicine, Medical Faculty and University Hospital Duesseldorf, Heinrich-Heine University, 40225 Duesseldorf, Germany; gereon.poschmann@hhu.de (G.P.); kai.stuehler@hhu.de (K.S.); 3 Molecular Proteomics Laboratory, Biomedical Research Centre (BMFZ), Heinrich-Heine-University, Universitätsstrasse 1, 40225 Duesseldorf, Germany

**Keywords:** infertility, receptivity, window of implantation, embryo, uterus

## Abstract

Endometrial scratching (ES) has been widely used in assisted reproductive technology to possibly improve pregnancy rates, but its exact mechanism is still not understood or investigated, and its benefits are controversially discussed. Hypothetically, ES may trigger a local immune response, leading to an improved endometrial receptivity. So far, it has been shown that ES affects the gene expression of cytokines, growth factors, and adhesive proteins, potentially modulating inflammatory pathways and adhesion molecule expression. Our pilot study applying proteomic analysis reveals that ES probably has an impact on the proteins involved in immune response pathways and cytoskeleton formation, which could potentially increase endometrial receptivity. Specifically, proteins that are involved in the immune response and cytoskeleton regulation showed a trend toward higher abundance after the first ES. On the other hand, proteins with a decreasing abundance after the first ES play roles in the regulation of the actin cytoskeleton and cellular processes such as intracellular transport, apoptosis, and autophagy. These trends in protein changes suggest that ES may affect endometrial tissue stiffness and extracellular matrix remodeling, potentially enhancing the embryos’ implantation. To our knowledge, this pilot study provides, for the first time, data investigating potential changes in the endometrium due to the scratching procedure that might explain its possible benefit for patients in infertility treatment. Furthermore, the proteome of a group of patients suffering from repeated implantation failure was compared to that of the fertile group in order to transfer the basic science to clinical routine and application.

## 1. Introduction

Endometrial scratching (ES) is a medical procedure that involves intentionally injuring the human endometrium, the inner lining of the uterus, in the luteal phase of the menstrual cycle [1]. The endometrium undergoes cyclic changes during the menstrual cycle under the influence of steroid hormones and during decidualization. The functional layer of the endometrium, which consists of epithelial and stromal cells, prepares for the implantation of an embryo. Successful implantation relies on the interaction between the embryo and the receptive endometrium, which is a complex tissue with various cell types and dynamic changes in gene expression, protein synthesis, and immune response [2,3]. Understanding its biology is crucial for infertility treatments and improving pregnancy outcomes. A fertile female typically has the potential to become pregnant during her window of implantation (WOI), which is the time after ovulation between days 19 and 23 of a regular 28-day menstrual cycle [4,5]. If applied, ES is performed on women undergoing assisted reproductive technology (ART), such as in vitro fertilization (IVF), with the aim of enhancing the implantation rate [2]. Even though ART success has improved, the success rates are still not satisfactory because about 70% of the procedures do not result in pregnancy [6]. The goal of injuring the endometrium is to promote endometrial receptivity, possibly by enhancing the population of immune cells required for a successful pregnancy [7]. Barash et al. demonstrated in 2003 for the first time that intentionally injuring the endometrium results in higher pregnancy rates [7]. Since then, this procedure has been highly controversially discussed in the literature but widely applied in infertility clinics, although the benefit has been questionable to date. In general, it is employed in patients with repeated implantation failures (RIF) [8,9]. Since RIF is a huge burden in ART, both patients and doctors were intrigued by the idea of improving the therapy outcome by approaching a simple and fast procedure such as ES [10]. Nevertheless, ES was not recommended for women undergoing their first IVF cycle but was supposed to be a tool for patients with multiple implantation failures [8,10,11]. From the initial reports on ES, the infertility community controversially assessed these results, and a common point of criticism was the fact that the physiology and subsequent changes of the endometrium executed by the procedure were and are still unclear [12,13]. However, ES is not only applied for the purpose of possibly enhancing pregnancy rates but also as a procedure to gain endometrial tissue for diagnostic investigations such as the detection of uterine natural killer cells (uNK), plasma cells (PC), and Endometrial Receptivity Analysis (ERA) [14].

Several hypotheses exist regarding the mechanism by which ES may improve pregnancy rates. First, ES is thought to trigger a healing response and/or an immune response in the endometrium. This healing process may lead to an increased release of growth factors, cytokines, and other molecules that promote a more favorable environment for embryo implantation [7]. Additionally, it is hypothesized that the injury stimulates a shift from a pro-inflammatory to an anti-inflammatory environment, reducing the likelihood of the immune rejection of the embryo [15]. There have been a few studies investigating the effects of ES on gene expression in the endometrium, and they show that the genes involved in immune signaling pathways were modulated [16]. Aghajanpour et al. showed that in RIF, innate immune-associated genes such as TLR3 and TLR4 were decreased. Further adaptive immune-related genes such as IFN γ, CD80, and CXCR8 showed reduced expression [16]. On the contrary, some studies did not find any advantages [17,18]. Furthermore, some papers that address this controversy even recommend not performing intentional ES due to a lack of convincing data and the obscureness of the physiology behind the scratching [12,19]. Simon et al. already criticized in 2014 that there was no consensus for the procedure itself, such as the degree of the injury, the exact cycle day, the time elapsed to the embryo transfer, and to which patient cohort it is beneficial [16]. By now, the effect of ES has been mainly analyzed in studies on patients who undergo infertility treatments. The limitations of those studies are the availability of patient samples and the inhomogeneity of the investigated groups. Particularly since, up to date, it is not clear what this intervention changes or induces in the endometrium, we analyzed for the first time the proteomic changes in fertile females 4 weeks after the initial procedure of ES during the WOI to perceive the underlying physiology, which possibly provides insight into why it may enhance pregnancy rates or not. In order to answer the question of a clinical application, the proteome data of a RIF group were additionally compared to the initial dataset of the fertile group (42).

## 2. Results

### 2.1. Proteomic Analysis of ES Samples Follow-Up vs. Initial Biopsy

**Figure 1:**
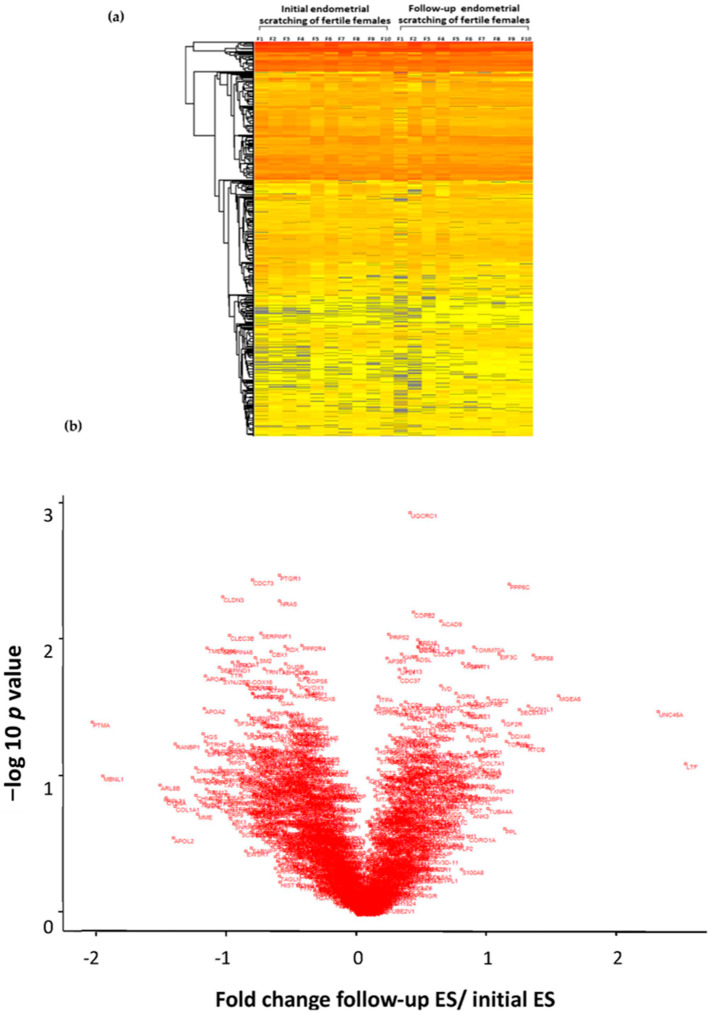
Clustering heat map showing the relative protein abundance pattern from low (yellow) to high (red). F1 to F10 display the fertile females (n = 10) (**a**). Volcano plot of the proteomic analysis showing the fold change (difference of group means of log_2_ normalized intensities) of follow-up endometrial scratching tissue 4 weeks after the initial ES (n = 10) vs. initial endometrial scratching tissue (n = 10) from fertile individuals. All identified proteins are shown with their associated gene names (**b**).

For this study, the proteome of the ES was analyzed from 10 fertile females and compared to a follow-up scratching 28 days later. The analysis of the proteome of the initial and follow-up ES samples identified several ontology groups comprising proteins that differed in their abundance between the initial and follow-up biopsy. In Figure 1, we present the proteome changes of the fertile females as a heat map and a volcano plot. The outlier dots represent proteins with a trend to be influenced in their abundance by ES and therefore might induce changes in the endometrium, possibly leading to enhanced receptivity in the subsequent cycle; nevertheless, due to correction for multiple testing and high inter-individual differences, no significantly different abundant single protein is reported.

### 2.2. ES-Evoked Changes in Protein Abundance of Fertile Females and RIF Patients

**Figure 2:**
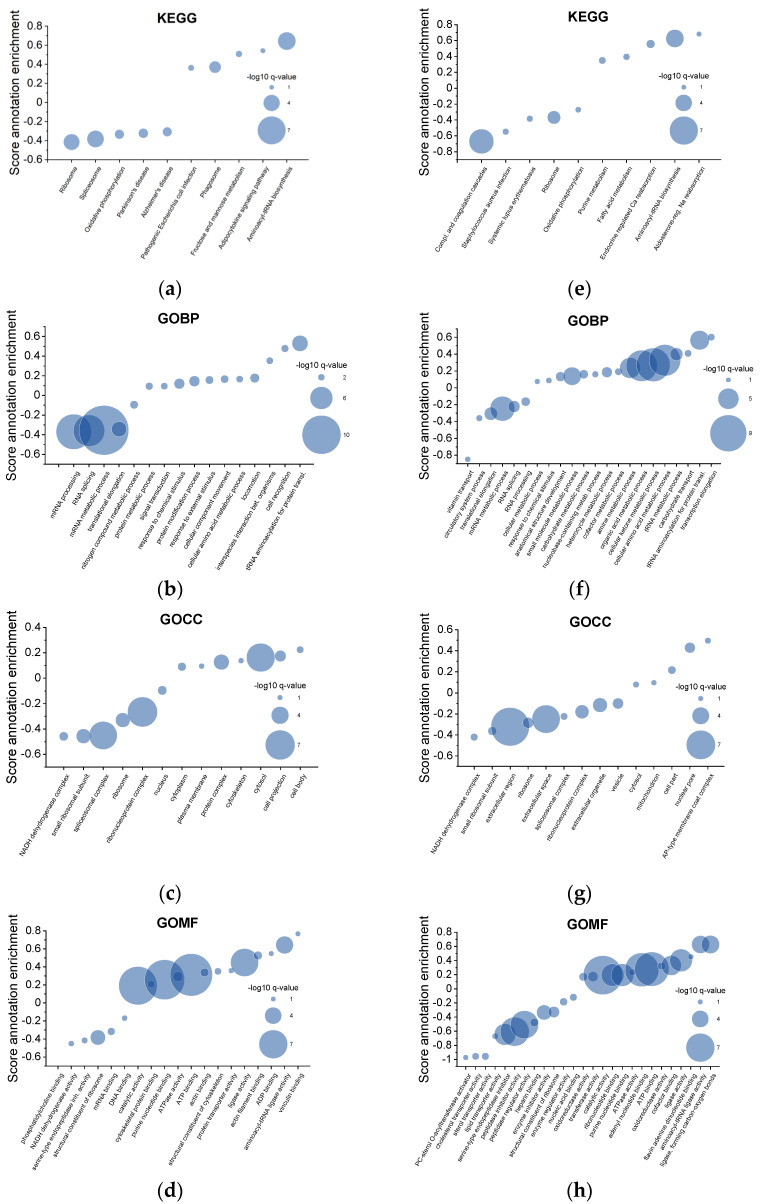
(**a**–**d**) One-dimensional annotation enrichment analysis of proteomic data from endometrial samples, follow-up versus initial scratching. (**e**–**h**) One-dimensional annotation enrichment analysis of proteomic data from initial endometrial samples from RIF patients and fertile females. Identified proteins were annotated with ontology annotation ((**a**,**e**) KEGG, (**b**,**f**) GOBP, (**c**,**g**) GOCC, (**d**,**h**) GOMF), and an annotation enrichment was carried out on differences in group means of log_2_-transformed mass spectrometric intensity values according to Cox et al., 2012 [20]. The presented score represents the distribution shift of protein abundances in a certain category relative to proteins not belonging to this category. Positive scores mean that there is a shift to higher abundances in samples after scratching and negative scores mean that there is a shift to lower abundances in samples after scratching. The log_10_ q-values represent the calculated log_10_ *p*-value which was corrected for multiple testing using the method of Benjamini and Hochberg. Presented categories have been selected to avoid redundancies. The complete list is available in the Supplementary Materials.

For the ontology annotation, Gene Ontology (GO) and Kyoto Encyclopedia of Genes and Genomes (KEGG) pathway enrichment analysis of the different protein abundances from the follow-up ES vs. initial ES groups were used (Figure 2a). The most enriched GO biological process (GOBP), cellular component (GOCC), and molecular function (GOMF) terms are indicated in Figure 2b–d. The most enriched KEGG pathways include phagosome and t-RNA synthesis (Figure 2a). The most affected GOBP categories comprising proteins shifted to higher abundances in the follow-up scratches include tRNA aminoacylation for protein translation, locomotion, protein modification processes, and the response to chemical stimuli (Figure 2b). The GOCC in Figure 2c shows enrichment in proteins belonging to the cytosol, cytoplasm, and plasma membrane and also in the cell projection, cell body, and cytoskeleton. The enriched GOMF are, among others, cytoskeleton-binding and actin-binding. The highest enriched functions are catalytic activity, purine nucleotide binding, ATP binding, ligase and aminoacyl-tRNA ligase activity (Figure 2d). Proteins associated with spliceosomal and ribosomal complexes showed a lower abundance, comparing the follow-up ES vs. the initial ES (Figure 2a–d). Most of the proteins, which showed a different abundance, belong to processes such as metabolism, immune response, protein transport, protein processing, and cytoskeleton organization. Further changes could be observed for proteins involved in the cell cycle, cytoplasm, and cell signaling.

In the study conducted by Bielfeld et al. in 2019, proteomic analyses on individuals suffering from Recurrent Implantation Failure (RIF) were performed. We compared these data to our initial dataset derived from fertile women. Interestingly, upon analyzing the RIF enrichment data, we uncovered some intriguing parallels in terms of protein abundance. Employing the KEGG analysis, we noted similar patterns between follow-up vs. initial ES (Figure 2a) and RIF vs. initial ES (Figure 2e), specifically observing a decrease in proteins associated with the ribosomal pathway, paired with an increase in proteins related to the aminoacyl-tRNA biosynthesis pathway (Figure 2a,e). Notably, a discernible divergence emerged when contrasting RIF and fertile female cohorts, characterized by a larger cohort with a diminished presence of proteins associated with the complement and coagulation cascades in the RIF group. The enrichment analysis of GOBP terms comparing the follow-up vs. initial samples (Figure 2b) and the RIF vs. initial ES (Figure 2f) looks different, with more identified pathways in the latter comparison (Figure 2f). The follow-up ES yielded larger cohorts of proteins belonging to RNA splicing and processing (Figure 2b), whereas the abundance of proteins involved in RNA processing was smaller in the RIF group compared to the fertile collective (Figure 2f). The RIF group showed exclusive variations in protein abundance in processes concerning the metabolism of organic acid, cellular ketone, and cellular amino acid metabolism, to name a few (Figure 2f). Turning to the GOCC annotation, we observed a consistency in protein abundance within structures, including the NADH dehydrogenase complex, small ribosomal subunit, spliceosome complex, ribosome ribonucleoprotein complex, and nucleus between the different groups (Figure 2c,g). Conversely, GOCC distinctions between RIF and fertile female groups exclusively showed processes in the extracellular region and space, with a lower abundance in the RIF group (Figure 2g). Moreover, when scrutinizing the GOMF analysis, we noticed congruencies in functions such as purine nucleotide binding, ATPase activity, and ATP binding, with heightened abundance (Figure 2d,h). This was juxtaposed with a diminished presence of serine-type endopeptidase inhibitor activity. Intriguingly, ligase activity and amino-acyl-tRNA ligase activity exhibited a reinvigorated abundance. Overall, the enrichment pattern underscored striking similarities within the realm of ribosome complexes and tRNA ligases, accompanied by their relevant pathways. Moreover, Figure 2h presents more protein abundances and molecular functions in the comparison of RIF vs. the fertile group than in the fertile group between the follow-up and initial samples, underscoring the differences in the RIF group (Figure 2d,h).

## 3. Discussion

ES was widely used in ART clinics after it was first described two decades ago, but its exact mechanisms are still unclear, the real benefit is doubtful, and therefore, ever since its introduction, it has been under investigation [9]. For the first time, we analyzed the effect of ES on protein levels in fertile females in two consecutive menstrual cycles during the WOI and identified which proteins and pathways are affected by the procedure. It has been discussed that ES may have an influence on the immune response and/or cell organization [7]. Previous studies showed that ES changes the gene expression of cytokines, growth and transcription factors, and adhesion proteins [10]. Furthermore, so-called pro-implantation proteins such as mucin 1 transmembrane (MUC1), crystallin alpha B, apolipoprotein D (APOD), phospholipase A2 (PLA2), and uroplakin Ib (UPIb) were altered in their gene expression [21]. By doing so, the receptivity of the endometrium is supposed to increase in the next cycle. Gnaisky et al. suggested that monocytes that were recruited to the scratched area remained for a longer period [22]. It is widely accepted that ES triggers the local immune response, which might lead to a better receptivity. Potdar et al. proposed applying the ES performed in a preceding cycle before ART treatment because these immune response modulating actions refining the endometrium’s structure need some time to fully flourish, being controlled by steroid hormones [10]. Dekel et al. hypothesized that ES enhances receptivity to a favorable status by triggering an inflammatory pathway, which changes the expression of adhesion molecules of the epithelial cells [23]. Further, Lia and Hao et al. showed that ES influenced the expression of the estrogen receptor in the preceding cycle [24].

During decidualization, a phenotype change occurs, especially through the reorganization of the actin cytoskeleton [14]. Our findings indicate that ES probably has an influence on the abundance of proteins involved in immune response pathways (Figure 2a,b) and in cytoskeleton formation (Figure 2c,d). These may increase endometrial receptivity in the subsequent cycle by modifying the maternal immune response locally in the endometrium and the cellular dynamics of endometrial cells. It is important to mention that although the overall fold changes are not very high, considering the limited availability of proteome data so far, it is intriguing to observe where trends could be identified and how they possibly contribute to the comprehensive picture of embryo implantation. In Figure 1b, the proteins are presented that have a fold change 1.5 times higher after the initial ES, namely lactoferrin (LTF), protein UNC-45 homolog A (UNC 45A), and O-GlcNAcase (MGEA5). These three proteins are involved in the immune response and cytoskeleton formation. LTF is a part of the neutrophil phagosome and acts as an antimicrobial granule during phagocytosis, a process that involves the detection and engulfing of pathogens [25]. It is known that LTF has a positive effect on the microbiome of the reproductive tract and shifts the endometrium to a more receptive state. It is even recommended to apply oral LTF supplementation to RIF patients [26]. Noticeably, Yanaihara et al. showed that LTF can stimulate the proliferation of stromal cells in vitro [27]. UNC-45A is a member of the protein family of co-chaperones and plays an important role in regulating cytoskeletal-associated functions. UNC-45A functions as a mitotic spindle-associated protein that destabilizes microtubule activity and was overexpressed in human ovarian and breast cancers, and its loss results in reduced cell proliferation [28]. In addition, MGEA-5 was shown in endometrial cancer cells to reorganize the cytoskeleton [29]. MGEA-5 and UNC-45A, as microtubule destabilizing proteins, may contribute to changing the cytoskeleton of the endometrial cells, which might result in different tissue stiffness and, therewith, possibly make it easier for the embryo to invade.

MBNL1, similarly to ARL8B.2 and Prothymosin Alpha (alpha PTMA), showed a decreasing trend after ES with fold changes higher than 1.5 (Figure 1b). MBNL’s function as an RNA-binding protein regulating RNA alternative splicing, localization, and integrity shows a trend of lower abundance [30,31]. Additionally, the annotation enrichment analysis shows a significantly lower protein abundance for RNA splicing and spliceosomal-complex-associated proteins between the follow-up ES and initial ES (Figure 2a–c). For example, a downregulation of MBNL1 may influence the transcriptome of endometrial cells and, therewith, may have an impact on their differentiation. For endometrial receptivity, the decidualization of stromal cells, which differentiate into a specific cell shape and secretion pattern, is crucial [32,33]. Arl8B is important for intracellular transport, especially for lysosome movement [33]. In recent studies, Arl8b has been associated with the remodeling of the extracellular matrix (ECM) and contributing to invasiveness [34]. During decidualization, the endometrial ECM is modified and therefore possibly contributes to endometrial receptivity [33,35]. As of today, there are limited data on MBNL1 and ARL8b and their role in endometrial receptivity; hence, further studies are needed.

The human prothymosin-α (PTMA) protein functions in the cytosol as an anti-apoptotic effector through the inhibition of apoptosome formation [36]. Extracellularly, it has an immunomodulatory effect [37,38]. In the context of reproduction, the fact that PTMA promotes the transcriptional activity of the estrogen receptor [39] is of interest. In several studies, it has been shown that PTMA seems to have an impact on the ECM of cells. For example, in fibrosis, PTMA is often increased, and a knockdown results in reduced protein levels of collagen I, α- smooth muscle actin (SMA), and matrix metalloproteinases (MMP) [40]. Hence, it is possible that PTMA is reduced after ES to soften the endometrial tissue, leading to an easier invasion for the potential embryo to be embedded in the endometrium. Interestingly, PTMA and NRAS were also identified before by our group in a study comparing the proteomic endometrial pattern of fertile females in comparison to patients suffering from RIF. Furthermore, in this study, it was shown that the proteome composition was affected for pathways such as metabolism and the immune system [41]. It is imaginable that these proteins may have aberrant regulation in RIF patients, and ES restores a more suitable expression of the proteins, which results in better receptivity and consequently better implantation rates. These findings indicate that PTMA and NRAS should be investigated further in the context of proper embryo implantation. Additionally, it was shown that apoptosis and autophagy play a role in implantation [42,43]. Interestingly, PTMA was recently stated to have a pro-autophagic role in human testis. PTMA possibly serves as a switch from apoptosis to autophagy [44]. Therefore, it could also be hypothesized that it links the crosstalk of apoptosis and autophagy in female reproduction. After ES, the lower abundance of proteins such as PTMA possibly results in enhanced apoptosis and autophagy, which boost tissue remodeling and promote the attachment and invasion of the embryo into the endometrium. In further studies, it would be interesting to investigate how apoptosis and autophagy are regulated during implantation with respect to PTMA and if patients with RIF have an aberrant regulation.

Furthermore, certain proteins showed a trend toward having a higher or lower abundance, and some are interesting to look closer at for their potential role in implantation. An increasing trend after ES was observed in signal recognition particle 68 (SRP68), SEC61A1, and serin-threonine-protein phosphatase, PP5C. SRP68 is involved in protein targeting and transport as part of the signal recognition particle complex. It plays a crucial role in delivering newly synthesized proteins to the appropriate cellular compartments [45]. Furthermore, it could be shown that it is overexpressed in endometrial cancer [46]. Particularly, the processes of embryo and tumor invasion can be compared [47]. SEC61A1 is the largest part of a protein complex associated with the membrane of the endoplasmic reticulum and is responsible for protein transport [48]. Salsano et al. have shown that SEC61A1 plays a role in decidualization. They found a novel progesterone receptor and concluded that SEC61A1 was implicated in the remodeling of endometrial cells during decidualization and aggregated with proteins involved in biosynthesis, intracellular transport, and mitochondrial activity [49]. PPP5C is known to be a regulator in the MAPK kinase pathway [50]. In the context of embryo implantation, the MAPK signaling pathway plays a critical role in regulating the interaction between the embryo and the receptive endometrium. It is involved in mediating the complex crosstalk between the embryo and the endometrial cells, facilitating embryo adhesion, invasion, and successful implantation [50]. The activation of the MAPK signaling pathway in the endometrium promotes various molecular and cellular changes necessary for embryo implantation, such as the remodeling of the endometrium, angiogenesis, and the establishment of a receptive environment for embryo attachment [51].

In our study, the fertile females showed a decreasing trend in apolipoprotein L2 (APOL2), apolipoprotein A2 (APOA2), and Ran binding protein 1 (RanBP1) after ES. APOL2 is known to have a role in decidualization [52]. In their study, Brosens et al. discovered that apoA-I, a protein secreted by the developing endometrium, could potentially hinder the process of embryo implantation. They proposed that disruptions or changes in the regulation or modification of apoA-I could be a crucial factor in the development of endometriosis and RIF. APOA2, like APOL2, belongs to lipoproteins and plays a role in fatty acid oxidation, lipid metabolism, and fat digestion [53]. Lipoproteins are generally known to be important for embryonic development. It was demonstrated that lipoproteins are part of the assembly of signaling factors and that the lipoprotein receptors are significant during embryonic patterning pathways [54]. Generally, lipoproteins are the main components of high-density lipoprotein (HDL) and low-density lipoprotein (LDL). APOA2 is a part of HDL, and HDL in follicular fluid had a negative impact on embryonic development in IVF culture in vitro [55]. Ran binding protein 1 (RanBP1) was decreased after ES. It is a cytoplasmic-enriched and nuclear-cytoplasmic shuttling protein, playing an important role in nuclear transport [56]. Recent studies have shown that RanBP1 controls spindle checkpoint formation and seems to be affected in some cancers [57]. Interestingly, Rensen et al. showed that downregulation of Ran BP1 in cancer cell lines resulted in enhanced apoptosis [58]. As mentioned before, apoptosis is crucial for embryonic implantation. Therefore, the down-regulation of Ran BP1 could promote embryo implantation through increased apoptosis induced by the intentional injury of the endometrium.

Proteins associated with aminoacyl-tRNA biosynthesis, especially proteins showing ligase activity with aminoacylation and ribosome complex proteins (Figure 2a–d), were found to be more abundant after ES. Interestingly, Park et al., 2020 found that a Tryptophan tRNA ligase was released at the site of an injury in endometrial stem cells and promoted cell growth, migration, and differentiation in a cytokine-like fashion [59]. The process of ribosomal biogenesis is strongly linked to protein synthesis, differentiation, and apoptosis. The main role of the ribosome is to produce proteins using mRNA as a template and amino acids as the essential components. The regulation of active ribosome biogenesis is dependent on the cell cycle and is commonly associated with cellular plasticity and de-differentiation. Prakash et al. showed that the biogenesis of the ribosome influences the EMT. Changes in ribosomal activity can affect the destiny of the cell, leading to diverse ribosome-related conditions such as cancer [60]. Therefore, our findings indicate that maybe the increasing aminoacetyl-tRNA biosynthesis and reduction in ribosome complexes interplay in an unknown fashion and result in systemic changes in the endometrium.

Proteins known in RNA metabolism may be affected initially in RIF, leading to a dysfunctional endometrium and ES changes, which lead to an increased susceptibility of the endometrium to RIF. Currently, our understanding of the role of tRNA synthetases in human fertility remains limited. The horizon beckons with prospects for a more comprehensive exploration of their functions within the context of the endometrium. Interestingly, in Figure 2e–h, the GO curves look similar in their pattern between RIF compared to initial fertile ES and RIF compared to the fertile group.

Therefore, ES may affect the same signaling pathways, cellular compartments, biological processes, and molecular functions in different abundances. The curves in Figure 2f,g look different from their fertile counterpart (Figure 2b,c) indicating differences in the RIF group. However, these are only trends in the protein patterns observed in a pilot study, and more data are needed to verify these results, but as a first study, it may reveal some protein signatures that give a first indication of what happens due to the endometrial scratch.

In summary, our observations suggest that after ES, proteins that contribute to structural changes, immune responses, and maybe apoptosis are modified and therefore might result in enhanced endometrial receptivity in the subsequent cycle. In the future, it is important to investigate the effect of ES in RIF patients and compare the data to the established proteome profile herein in fertile individuals to understand the impact of this procedure for assisted reproductive therapies.

## 4. Materials and Methods

### 4.1. Identification of Fertile Females (FF) and RIF Patients

Endometrial tissues were collected through endometrial biopsies performed on days 19 to 23 of the menstrual cycle. The timing of the biopsies was determined based on the LH (luteinizing hormone) surge plus 7 days. A total of 10 individuals (FF), aged between 35 and 43 years, who had experienced at least one and up to three live births after natural conception, were recruited for the study. Six RIF patients were included with at least 3 unsuccessful ETs with good quality embryos (age 32–43 years). During ES, a thin, flexible catheter or biopsy instrument was inserted through the cervix into the uterus. The instrument was then moved gently along the inner surface of the endometrium to create small abrasions. The procedure is usually performed in an outpatient setting [2,13]. The research was carried out in compliance with the principles stated in the Declaration of Helsinki and received approval from the Ethics Committee of Heinrich-Heine University Düsseldorf (5528R [2016-06-16], 4394R [2016-05-24]). All participants provided written consent before taking part in the study.

### 4.2. Extraction, Identification, and Quantification of Proteins Using Mass Spectrometry

The extraction and analysis of the proteins from the endometrial biopsies were performed as previously described by our group [42]. Briefly, endometrial biopsy samples were collected and placed in a DMEM-F12 medium supplemented with HEPES, penicillin/streptomycin, and amphotericin B (all Biowest, Nuaille, France). Protein lysates from endometrial tissue were prepared and processed for mass spectrometric analysis (Thermo Scientific, Dreieich, Germany). Proteins and peptides were identified and quantified, and data analysis was performed within the MaxQuant Software (version 1.5.7.0, MPI for Biochemistry, Planegg, Germany) [61].

### 4.3. Statistical Analyses

For the determination of the fold change and *p*-values, the method was used as described by our group before [42]. Protein intensities from label-free quantification were analyzed using the Perseus software (version 1.5.8.5, MPI for Biochemistry). Clustering and group comparisons between the initial and follow-up scratches were conducted considering proteins with at least 8 valid values in at least one sample group. Missing values were estimated, and statistical significance was determined using *t*-tests and the significance analysis of microarrays method [62]. This method includes a control/correction for multiple testing based of a false discovery rate of 5%. Moreover, abundance shifts of functional related proteins were detected by one-dimensional annotation enrichment analysis. [20] This method detects functional- or ontology-based protein clusters that significantly differ in their intensity distribution to higher or lower abundances between the two sample groups. As control for multiple testing, the common method described by Benjamini and Hochberg was applied.

## 5. Conclusions

In this study, we have shown for the first time that ES evoked changes in the proteome of fertile females by examining initial and follow-up samples taken within four weeks. RIF patients showed similarities as well as differences in protein abundances and processes when comparing the initial samples of RIF and fertile women. The pathways and processes involving cytoskeleton, immune response, and metabolism are the most affected ones. The cytoskeleton facilitates cellular dynamics and embryo adhesion and invasion, the immune response maintains immune tolerance and regulates tissue remodeling, and the metabolic changes support the nutrient demands of the embryo. These three processes are probably connected and may facilitate successful embryo implantation. To date, most of the limited data on the endometrial changes induced by ES have been examined on the genome or RNA level, with very little on the protein level. However, the protein level is more interesting since proteins affect tissue behavior and constitution. Moreover, our findings show how important it is to investigate the effect of a procedure such local endometrial scratching to better understand the possible mechanism of action before a procedure is introduced into daily clinical practice.

## Data Availability

The data presented in this study are available in the Supplementary Materials.

## Supplementary Materials

The following supporting information can be downloaded at: https://www.mdpi.com/article/10.3390/ijms241713577/s1.
